# Molecular mechanisms of plant hormones and sugars regulates strawberry fruit development and ripening

**DOI:** 10.1007/s11103-026-01714-w

**Published:** 2026-05-11

**Authors:** Calistene Aparecida Pinto, Helyemari Valentim Althaus, Ranyelly Leão Coutrim, Ricardo Antonio Ayub, Fernanda Grimaldi

**Affiliations:** 1 Department of Plant Science and Phytosanitary, State University of Ponta Grossa, Ponta Grossa, Paraná 84030-900 Brazil; 2 Department of Plant Science and Phytosanitary, Federal University of Espirito Santo, Alegre, Espírito Santo 29500-000 Brazil; 3 Social Service of Industry (SESI), Regional Department of the State of Santa Catarina, Lages, Santa Catarina 89520-000 Brazil

**Keywords:** Hormonal crosstalk, Maturation, Young fruit, Gene expression, *Fragaria* spp.

## Abstract

The strawberry consists of two main parts: the dry achenes (the true fruit) and an enlarged receptacle (the fleshy part). These parts exhibit high metabolic synchrony during the development and ripening stages. Due to the low rates of ethylene production and respiration during ripening, strawberries are currently classified as non-climacteric fruits. However, studies suggest that ethylene, along with its interactions with other plant growth regulators like abscisic acid and auxin influences the ripening process in strawberries. Auxins are crucial for the initial development of fruits, promoting cell expansion and modulating non-climacteric ripening. Recent research indicates that cytokinins and gibberellins might play a role in enhancing fruit development and ripening by promoting cell expansion. Additionally, the literature suggests that increased sugar concentration, accompanied by cell wall degradation and changes in pulp osmotic pressure, is linked to hormonal crosstalk between growth promoters, abscisic acid and ethylene. Although traditionally classified as non-climacteric, recent evidence may suggest that strawberries operate under a model similar to ‘suppressed climacteric’. In this system, ethylene does not act as the primary trigger, but rather as an essential modulator of late ripening. Thus, strawberry ripening reflects a sophisticated hormonal interaction essential to the strawberry ripening cascade.

## Key Message

Strawberry ripening is regulated by coordinated hormonal crosstalk involving auxin, abscisic acid, cytokinins, gibberellins, and ethylene, suggesting an alternative ripening model in which ethylene functions as a late-stage modulator rather than the primary trigger.

## Introduction

Strawberry is one of the most relevant productive species of the Rosaceae family (Folta and Davis 2006; Husaini and Neri 2016). Composing the group of berries, in 2024 the world production of this fruit was estimated at 10.728 million tons in approximately 436 thousand hectares area (Faostat 2026). The strawberry is characterized as a pseudo fruit, formed by the fleshy receptacle and its true fruits, denominated achenes (Shamaila et al. 1992; Chandler et al. 2012).

Fruit ripening, including that of strawberries, is a highly complex and tightly regulated process that involves changes in color, texture, aroma, and flavor, resulting from the combined action of biochemical compounds such as hormonal control, which determine chemical, physiological, and molecular changes and are influenced by environmental signals and cross-interactions among various plant hormones (Shakya and Lal 2018; Pérez-Llorca et al. 2019). In contrast, two major modes of fruit ripening have currently been described in the literature, which differ mainly with respect to ethylene (ET) production and cellular respiration. These two physiological classifications are referred to as climacteric and non-climacteric fruits (Fuentes et al. 2019; Upadhyay et al. 2023).

Non-climacteric fruits are characterized by a decline in cellular respiration and a consistent rate of ethylene production during ripening, with abscisic acid (ABA) considered the key hormone regulating this process. In contrast, climacteric fruits exhibit high rates of cellular respiration and ET production, which peak during ripening and decrease upon full maturation (Fuentes et al. 2019; Perotti et al. 2023). Furthermore, studies have suggested that additional factors can influence fruit development, such as the interaction among other hormones and the accumulation of sugars, for example, determining molecular changes between these two models (Durán-Soria et al. 2020; Perotti et al. 2023).

However, a new approach classifies some fruits as suppressed climacteric, which exhibit ripening after harvest. For example, certain plum cultivars are characterized by very low ethylene production and a reduced respiratory peak, resulting in slow ripening both while still attached to the mother plant and after harvest (Huang et al. 2025).

Strawberry is widely recognized as a non-climacteric fruit and considered a model for this type of ripening. However, ET also participates in this process by regulating the expression of genes associated with pulp softening and interacting with ABA, stimulating its biosynthesis. Thus, ET contributes indirectly to strawberry ripening by reinforcing the hormonal signals that coordinate this process (Reis et al. 2020; Sun et al. 2013; Paul et al. 2011).

Although strawberry has traditionally been classified as a non-climacteric fruit and considered a model for this type of ripening, recent evidence suggests that it may not fit perfectly within this classic pattern. The involvement of ET in regulating genes related to pulp softening and in stimulating ABA biosynthesis indicates that strawberry exhibits intermediate characteristics, approaching a “non-classical non-climacteric” or partially climacteric fruit, similar to the concept of suppressed climacteric fruits.

In contrast, hormones such as auxin (IAA), cytokinin (CK), and gibberellin (GA) have been extensively studied during the early stages of fruit development, along with enzymes involved in cell wall synthesis. These factors exhibit a complex hormonal crosstalk with the hormones implicated in non-climacteric ripening processes (Li et al. 2016; Castro et al. 2021a).

In this review, we will discuss how hormonal crosstalk can influence ripening and its implications for understanding the metabolism of cultivated strawberry (*Fragaria* × *ananassa*) and its wild progenitor (*Fragaria vesca*). We will also address a new nuance in strawberry ripening, exploring evidence that it may exhibit intermediate characteristics between climacteric and non-climacteric fruits, with ethylene and abscisic acid participating in the regulation of the process, thereby broadening our understanding of the hormonal mechanisms involved and their applications in fruit production and quality.

### Strawberry fruit growth and development physiology

Strawberry development is a highly coordinated process involving functional interactions between the receptacle and the achenes, as well as a complex hormonal network. Morphologically, what is commonly referred to as the strawberry fruit is, in fact, a fleshy receptacle that turns red upon ripening, accumulates sucrose, and encloses the true fruits, the achenes. Fruit growth is highly dependent on communication between the achenes and the receptacle, with this interaction being essential both for maintaining early growth and for determining the final fruit size (Nitsch et al., 1950; Jia et al. 2013b).

**Fig. 1:**
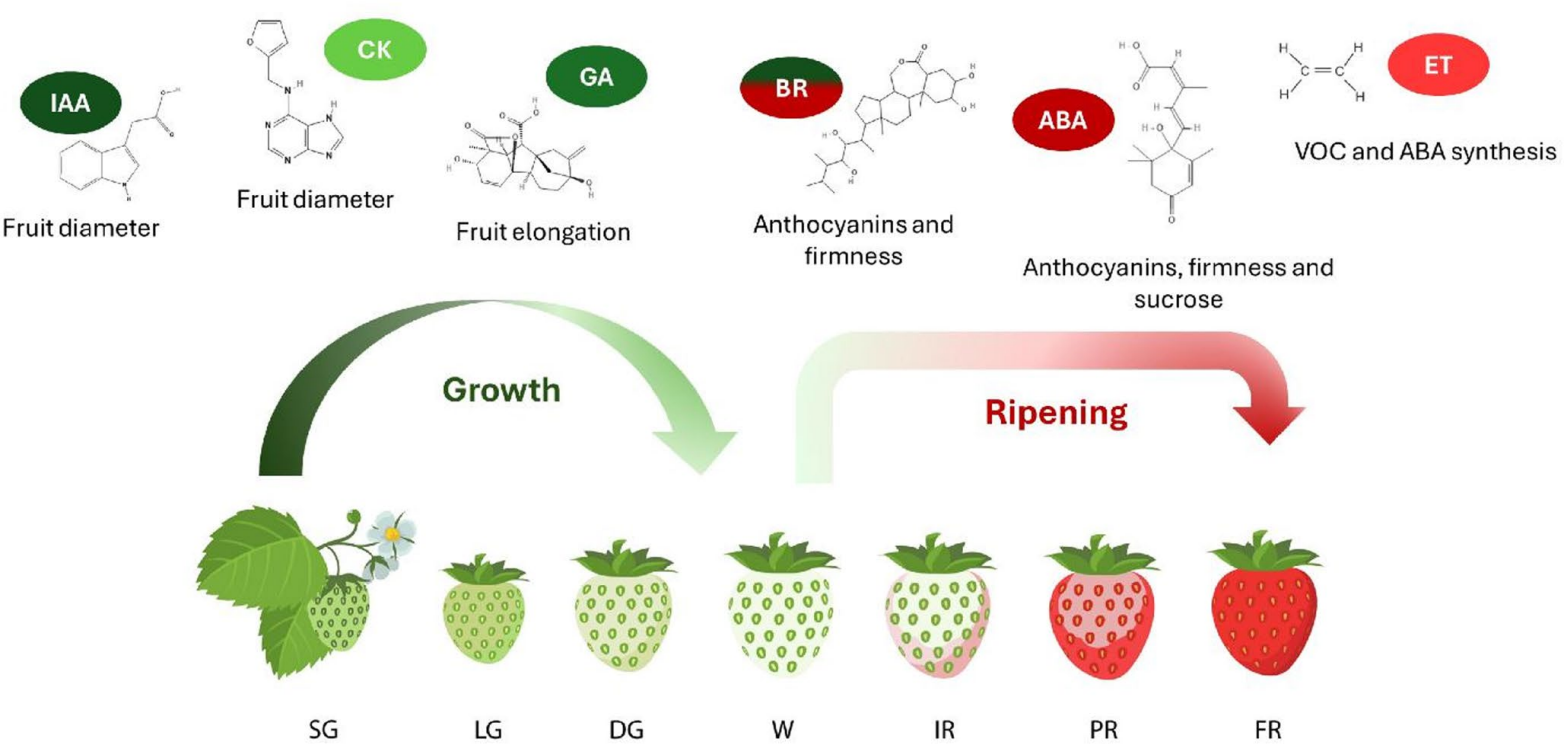
Plant hormones involved in the development and ripening phases of *Fragaria* spp. receptacles. IAA—indoleacetic acid; CK—cytokinin; GA—gibberellin; BR—brassinosteroids; ABA—abscisic acid; ET—ethylene; VOC—volatile organic compounds; SG—small green; LG—large green; DG—degreening; W—white; IR—initial red; PR—partial red; FR—full red. More intense shades of green indicate greater hormonal importance in fruit growth. More intense shades of red indicate greater hormonal importance in ripening

During the early stages after anthesis, identified as SG (small green) and LG (large green) in Fig. 1, strawberry development is characterized by intense mitotic activity, particularly in the receptacle, which functions as a significant metabolic sink. At this stage, fruit growth occurs predominantly through cell division, accompanied by the establishment of a functional vascular system that supports the transport of photoassimilates from source tissues. As the fruit develops, there is a gradual transition from cell division to cell expansion as the primary driver of growth, involving increases in vacuolar volume, cytoskeletal reorganization, and structural modifications of the cell wall (Liu et al. 2010; Feng et al. 2018).

Cell expansion-driven growth in the receptacle, particularly during the DG (degreening) and W (white) stages, is associated with changes in cell wall plasticity, osmotic potential, and turgor pressure, allowing cell volume to increase without compromising tissue integrity. During this phase, a progressive accumulation of soluble carbohydrates and an intensification of respiratory metabolism are also observed, reflecting the high energy demand required to sustain active fruit growth (Perotti et al. 2023).

Achenes play a central role as physiologically active centers during early development, functioning not only as reproductive organs but also as modulators of receptacle growth, influencing the spatial development pattern and fruit symmetry. Removal or damage to the achenes interrupts receptacle growth, highlighting the functional dependence between these structures (Nitsch 1950; Jia et al. 2020; Yang et al. 2025). As the fruit approaches the intermediate and final developmental stages, fresh mass accumulation slows, reflecting the approach to final size. At this stage, important physiological changes precede ripening, including reorganization of primary metabolism, modulation of water status, and preparation of the tissue for processes such as softening, pigment accumulation, and alterations in the volatile compound profile. Growth and ripening are, therefore, sequential yet physiologically distinct processes (Jia et al. 2011; Perotti et al. 2023).

Completion of growth does not immediately trigger ripening, and the interval between these events involves metabolic and structural adjustments that define the fruit’s ability to respond to endogenous and environmental signals leading to maturation. In summary, strawberry development is a classic example of interorgan and hormonal coordination, in which interactions between achenes and the receptacle, cell division and expansion, carbohydrate accumulation, and hormonal crosstalk provide the foundation for robust growth and subsequent fruit ripening.

### Hormonal coordination of early fruit growth

Strawberry fruit development is strongly regulated by a complex hormonal crosstalk, in which auxin, gibberellins, and cytokinins act in an integrated manner with various biochemical compounds. The interaction between these hormones is considered a central factor for fruit cell expansion, directly influencing its growth, shape, and ripening (Fuentes et al. 2019; Kumar et al. 2014).

Auxin, especially IAA, plays a predominant role in this process. High IAA concentrations in fertilized achenes are essential for normal receptacle growth, while the removal of achenes results in the cessation of this development. Although fruits treated with synthetic auxins are able to develop, they exhibit reduced anthocyanin synthesis, higher firmness, and elevated chlorophyll levels characteristics associated with delayed ripening (Nitsch 1950; Jia et al. 2020; Yang et al. 2025). Fertilized achenes constitute the primary auxin source, establishing hormonal gradients that promote intense cell division during early developmental stages and, subsequently, the expansion of receptacle cortex cells (Kang et al. 2013; Jang et al. 2024). This growth occurs predominantly in the radial direction, reflecting the local action of auxin in regulating cell wall plasticity, osmotic potential, and cytoskeleton organization—factors that favor lateral cell expansion (Liao et al. 2018; Yang et al. 2025).

Several genes involved in strawberry ripening are modulated by auxin, including those associated with its metabolism, pigmentation, aromatic compound biosynthesis, stress and defense responses, cell wall metabolism, and fatty acid metabolism (Chen et al. 2015; Estrada-Johnson et al. 2017; Jia et al. 2020). Genes of the main auxin biosynthetic pathway, TAA/YUC, show high expression during early fruit development stages. YUCCA family isoforms, such as *FaYUC1* and *FaYUC2*, reach transcription peaks at the small green fruit (SG) stage, with a progressive reduction starting from the white receptacle with green achenes (W) stage (Liu et al. 2010). In octoploid strawberries, *FaYUC10* and *FaYUC11* maintain sustained expression from the SG stage until the reproductive transition (TR) stage, while *FaYUC4* shows higher expression at the mid-green (MG) stage, indicating differentiated functions of these isoforms in maintaining auxin homeostasis (Jang et al. 2024). Functional evidence reinforces the importance of auxin in fruit growth. CRISPR/Cas9 gene editing of the *FveYUC10* gene results in a significant reduction of free auxin levels in developing fruits, compromising their growth and confirming the essential role of the TAA/YUC pathway in IAA synthesis (Mashiguchi et al. 2011; Feng et al. 2018).

**Fig. 2:**
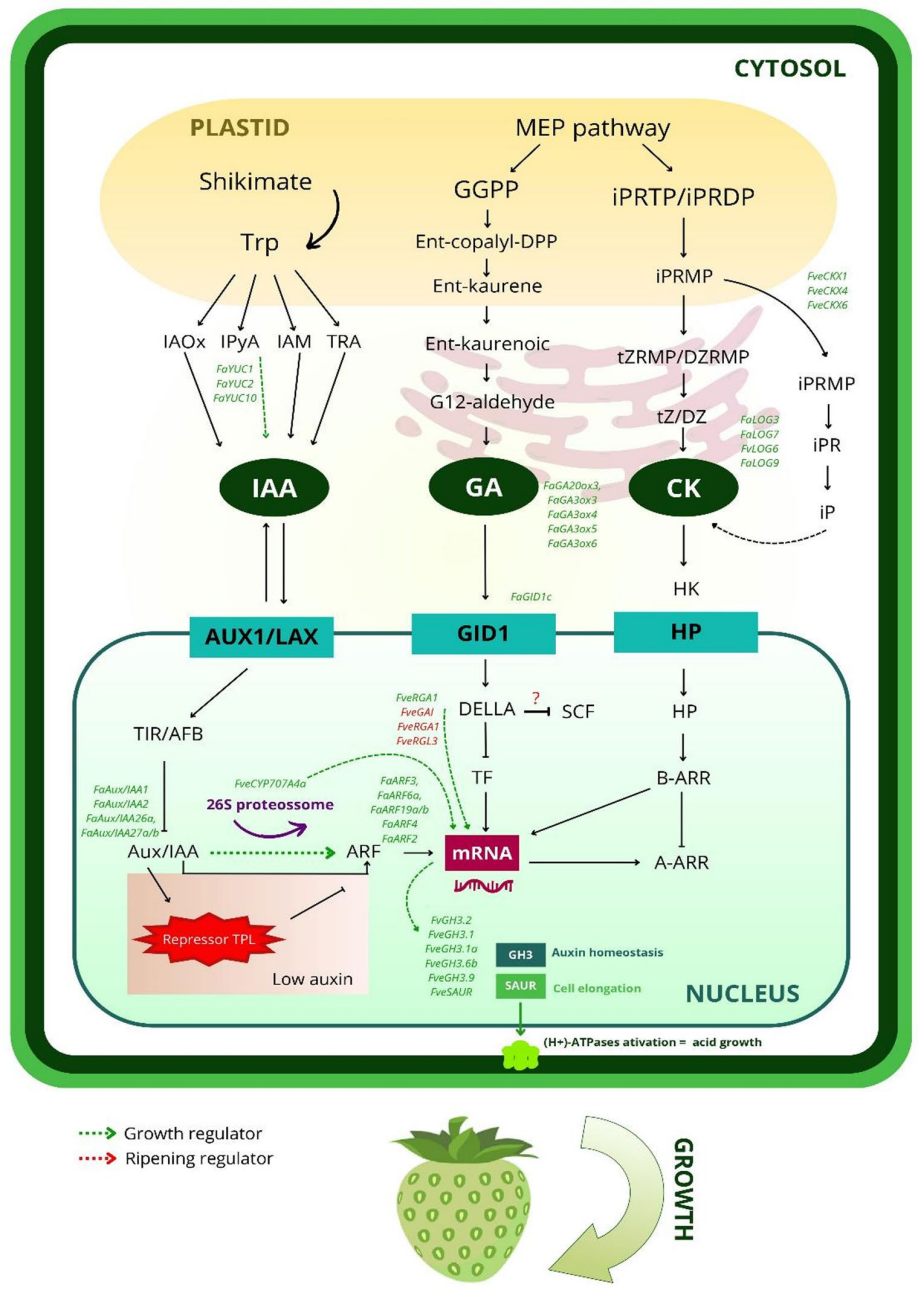
Biosynthetic and hormonal signaling pathways involved in the regulation of strawberry fruit growth. The biosynthesis and signaling of auxin (indole-3-acetic acid, IAA), gibberellin (GA), and cytokinin (CK) are depicted, highlighting the main precursors, enzymes, and regulatory genes identified in *Fragaria vesca*. Black arrows indicate biosynthetic routes or activation, while red lines indicate repression. The auxin influx carriers AUX1/LAX, receptors GID1 (Gibberellin Insensitive Dwarf1) and HP (Histidine-containing Phosphotransfer protein), and regulators such as TIR/AFB (Transport Inhibitor Response 1 / Auxin Signaling F-box proteins), ARF (Auxin Response Factor), TPL (TOPLESS), SCF (SKP1–Cullin–F-box protein complex), and DELLA (GA signaling repressors) coordinate transcriptional responses. Genes related to auxin homeostasis (GH3) and cell elongation (SAUR, *Small Auxin Up RNA*) are activated by ARFs. The activation of H⁺-ATPase proton pumps promotes acid growth and cell expansion, contributing to fruit development. HK (Histidine Kinase) and ARRs (Arabidopsis Response Regulators, type A and B) mediate cytokinin signal transduction

Auxin signaling in strawberries is primarily mediated by auxin response factors (ARFs) and Aux/IAA proteins, which regulate gene transcription in a development stage-dependent manner. During non-climacteric strawberry ripening, ARFs are expressed in both the achenes and the receptacle, showing low levels in green fruits and higher expression in red fruits (Estrada-Johnson et al. 2017). In contrast, *FaAux/IAA1* and *FaAux/IAA2* exhibit high transcription levels in early stages, with a progressive decline throughout ripening (Fig. 2) (Liu et al. 2010). This pattern reflects the classical auxin signaling model, in which ARFs bind to cis-responsive elements (AuxREs), while Aux/IAA proteins modulate their activity via protein–protein interactions, resulting in transcriptional activation or repression (Calderon-Villalobos et al. 2010; Jing et al. 2024).

Although most ARFs are more highly expressed in immature fruits, specific genes such as *FaTAR2, FaAux/IAA11, FaAux/IAA14b, FaAux/IAA33, FaARF6a,* and *FaARF16c* show higher expression in mature fruits, suggesting participation in ripening that is partially independent of auxin (Gu et al. 2019; Estrada-Johnson et al. 2017). Additionally, genes such as *FaAux/IAA26a*, *FaAux/IAA27a/b*, *FaTIR1*, *FaAFB2*, *FaAFB5*, and ARFs (*FaARF3, FaARF6a, FaARF19a/b*) are more highly expressed in the receptacle during the SG and MG stages, being associated with variation in fruit size and shape (Jang et al. 2024).

Recent studies highlight the role of *FaARF2* as a central link between auxin and abscisic acid (ABA) in controlling strawberry ripening. The overexpression of *FaARF2* reduces the expression of *FaNCED1* (a key enzyme in ABA synthesis) and ABA levels, delaying ripening, while its silencing increases ABA levels and accelerates the process, intensifying coloration, aroma, and sugar accumulation. Exogenous ABA application partially reverses the effects of *FaARF2* overexpression, evidencing the functional interaction between the auxin and ABA pathways (Li et al. 2023). Thus, high auxin levels in early stages induce *FaARF2* expression, repressing ABA, while the reduction of auxin allows for the activation of ripening mechanisms.

Auxin homeostasis is also regulated by genes of the GH3 and SAUR families. GH3 proteins catalyze the conjugation of IAA to amino acids, promoting its degradation or temporary storage (Staswick et al. 2005). In strawberries, genes such as *FveGH3.1*, *FveGH3.1a, FveGH3.6b,* and *FveGH3.9* are more highly expressed in the achenes and during early developmental stages, while FaGH3.2 shows high expression in the receptacle between the SG and large green (LG) stages, suggesting a negative feedback mechanism for reducing auxin levels at the MG stage (Jang et al. 2024; Jia et al. 2013b). SAUR family genes regulate cell expansion through the activation of plasma membrane H⁺-ATPases, promoting cell wall acidification and radial tissue growth (Spartz et al. 2012; Li et al. 2021).

In contrast to auxin, the biologically active gibberellins GA₃, GA₁, and GA₄ are present in both the achenes and the receptacle, especially in the early stages of development (Mesejo et al. 2016). Transcriptomic data indicate that auxin biosynthesis genes (*FaYUC5, YUC11, TAR1*) and gibberellin biosynthesis genes (*GA20ox3, GA3ox3—6*) are predominantly expressed in the achenes, while hormonal signaling components are more highly expressed in the receptacle, reinforcing the role of the achenes as the hormonal source and the receptacle as the responsive tissue (Kang et al. 2013; Perotti et al. 2023).

The application of gibberellin (GA₃) does not alter the coloration of pollinated large green fruits but promotes receptacle cell expansion at the white fruit stage, associated with increased GA₄ and the induction of genes such as *FaGA3ox* and *FaGID1c* (Umemura et al. 2023). Furthermore, fruits treated with auxin show increased expression of gibberellin biosynthesis genes (GA3ox and GA20ox) and repression of negative regulators of GA signaling (GAI, RGA1, and RGL3), indicating a synergistic interaction between these hormonal pathways (Perotti et al. 2023).

Although many auxin mechanisms in *Fragaria* × *ananassa* ripening are still poorly documented, studies in Fragaria vesca indicate strong molecular interaction between auxin and gibberellin. An example is the interaction between *FveRGA1* (a DELLA protein) and *FveARF8*, mediated by the C-terminal regions of both proteins, promoting hormonal crosstalk (Zhou et al. 2020). Other studies demonstrate an antagonistic effect between DELLA proteins (*FveGAI*, *FveRGA1*, and *FveRGL3*) and auxin activity, observed after exogenous treatment with NAA (Liao et al. 2018; Gu et al. 2019).

While auxin promotes radial growth and increased fruit diameter, gibberellins are primarily associated with cell elongation and axial growth. The action of gibberellins results in anisotropic expansion, associated with cytoskeleton reorganization and the oriented deposition of cellulose microfibrils, producing more elongated fruits. Consistently, fruits treated with gibberellin exhibit an elongated shape, whereas auxin treatments result in rounder fruits (Castro et al. 2021a; Lee et al. 2020).

Another classic axis of hormonal interaction occurs between gibberellins and ABA. Both hormones share the precursor geranylgeranyl diphosphate (GGPP), which leads to metabolic competition and crosstalk. This interaction favors GA and IAA during initial growth, maintaining low ABA levels. Liao et al. (2018) demonstrated that auxin and GA induce the expression of *FveCYP707A4a*, reducing endogenous ABA content during the growth phase. Upon the reduction of auxin and GA levels, ABA biosynthesis is activated, which inhibits IAA production and triggers ripening.

Finally, cytokinin also participates in strawberry fruit development, although its role is less understood. This phytohormone acts mainly on cell division and apical dominance, in addition to influencing floral differentiation and parthenocarpy. Transcriptomic analyses indicate an increase in the expression of genes related to the synthesis of trans-zeatin (tZ) and isopentenyladenine (iP) during the pre-ripening and full ripening stages, accompanied by a reduction in hormonal signaling (Gu et al. 2019). Isoforms of the LOG enzyme, such as LOG3 and LOG7, act in cytokinin activation during fruit development, while cytokinin oxidase genes (*FveCKX1, FveCKX4*, and *FveCKX6*) show higher expression in immature fruits, suggesting crosstalk with ABA (Jiang et al. 2016; Li et al. 2016). Genes involved in signal transduction, such as AHK2/3/4, AHP, and ARR, are also more highly expressed in immature fruits, promoting cell division while delaying ripening.

In general, available data indicate that auxin, gibberellins, and cytokinins act in an integrated manner during early strawberry fruit development, promoting growth and cell expansion while exerting antagonistic effects on ripening. A detailed understanding of these hormonal interactions still requires further investigation, but it is evident that the balance between these regulators is a determining factor for the final size, shape, and quality of the fruit.

### Fruit ripening regulated by ABA and sugars

Sugars play a crucial role in the maturation and post-harvest quality of fruits, being a key factor in consumer preference due to their contribution to osmotic adjustment, energy provision, and sweetness (Olmo et al. 2020; Schwieterman et al 2014). In the early stages of strawberry fruit development, the primary monosaccharides present are glucose and polyols such as mannitol, erythritol, and sorbitol. The main storage carbohydrate is sucrose, a photoassimilate responsible for the sweet taste of the mature receptacle. Although present in lower concentrations, other soluble oligosaccharides like galactose, myo-inositol, raffinose, and stachyose are also translocated by the phloem and can serve as carbon sources for sucrose synthesis (Durán-Soria et al. 2020; Jia et al. 2013a, 2016; Olmo et al. 2020). Sucrose accumulation begins approximately 20 days after anthesis (green stage) and increases significantly until full ripening (31 DAA), while fructose and glucose concentrations stabilize around 27 DAA and decrease at harvest time (Durán-Soria et al. 2020; Gu et al. 2019).

Strawberry sugar content varies according to environmental factors such as salinity, nutrient availability, shading, and available leaf area, and especially with genotype. In addition to environmental factors, hormones may play a key role in the availability and biosynthesis of sugars (Sutsawat et al 2008). Auxin, as previously mentioned, induces early fruit growth, and a crosstalk mechanism between this phytohormone and sucrose may occur during maturation. This delay is caused by the inhibition of the synthesis of cell wall degradation enzymes (modification and degradation of pectins) by auxin, which consequently reduces the transport and deposition of saccharides, delaying ripening (Dal Santo et al. 2020). Conversely, when sucrose is converted into fructose and glucose soon after being synthesized by the photosynthetic process, these sugars can regulate gene expression or participate in cell wall degradation metabolism. This possibly affects pectin through the high expression of transcripts encoding pectinesterase (PME) and polygalacturonase (PG), contributing to ripening (Luo et al. 2019).

Sucrose has been suggested to regulate strawberry ripening through interaction with both ABA-dependent and independent pathways. ABA has been identified as one of the primary plant hormones involved in fruit ripening, interacting with ethylene in climacteric fruits and serving as a key regulator in non-climacteric maturation (Li et al. 2023). Strawberry fruits treated with exogenous sucrose and ABA exhibited increased expression levels of ABA biosynthesis genes (*NCED2*), ABA signaling regulators (*SnRK2.2*), sucrose synthase (*SS*), and genes related to cell wall degradation metabolism (Cellulase 1—*CEL1* and Cellulase 2—*CEL2*) (Luo et al. 2020b) (Table 1).

**Table 1:** Main gene and proteins involved in strawberry receptacle ripening

Gene/Protein	Function	Metabolic function	Expression stage	References
*FaNCED1* *FaNCED2*	Catalyzes the cleavage of carotenoids, leading to ABA biosynthesis	Increases ABA levels, accelerating ripening by affecting sugar accumulation and cell wall degradation	Ripe fruits	Jia et al. (2013a, b); Li et al. (2023)
*FaBG1*	Encodes β-glucosidase, involved in the hydrolysis of ABA glucose ester to release active ABA	Facilitates ABA synthesis, affecting ripening	Transition and ripe fruits	Jia et al. (2013a, b)
*FaPYR/PYL/RCARs*	ABA initiates signal transduction	Interacts with PP2C to activate ABA-responsive genes, regulating ripening processes	Fruit development	Li et al. (2023)
*FaSnRK2.2*	ABA signaling pathway	Activates ABA-responsive genes	Ripe fruits	Luo et al. (2020a, b)
*FaSUS1, FaSUS3, FaSUS4*	Sucrose synthases	Sucrose synthesis and cell wall metabolism	Ripe fruits	Luo et al. (2020a, b)
*FaSUT1*	Sucrose transporter	Enhances sucrose uptake, influencing sugar accumulation and maturation	Transition and ripe fruits	Jia et al. (2013a, b)
*FaEXP1*	Cell wall loosening	Cell wall expansion	Ripe fruits	Luo et al. (2020a, b)
*FaPL*	Pectate lyase	Degrades pectin, promoting cell wall breakdown and ripening	Ripe fruits	Luo et al. (2020a, b)
*FaPG*	Pectin degradation	Breaks down pectin in the cell wall, aiding in fruit softening	Ripe fruits	Luo et al. (2020a, b)
*FaCHS, FaCHI, FaF3H*	Flavonoid biosynthesis	Anthocyanin and flavonoid synthesis	Ripe fruits	Luo et al. (2020a, b)
*FaETR2, FaETR13*	Ethylene initial signal transduction	Ethylene signaling, affecting ABA biosynthesis and ripening regulation	Green fruits	Pattyn et al. (2021); Elmi et al. (2016)
*FaEIN2, FaEIN7*	Positive regulators in the ethylene signaling pathway	Mediate ethylene signaling, influencing ABA accumulation and ripening processes	Green fruits	Pattyn et al. (2021); Elmi et al. (2016)
*FaERF118*	Repressor of ripening	Repressor of ripening	Green fruits	Zhang et al. (2022)
*FaERF316*	Positive regulators in the ethylene signaling pathway	Mediate ethylene signaling, influencing ABA accumulation and ripening processes	Ripening fruits	Zhang et al. (2022)
ARS	ABA-stress ripening transcription factor	Binds hexose transporter promoter, activating sucrose-responsive genes and inducing ripening	Ripening fruits	Jia et al. (2016); Siebeneichler et al. (2020)
SAMS, ACS, ACO	Ethylene biosynthesis	Convert methionine to ethylene, influencing ABA biosynthesis and cell wall modification	Green and ripe fruits	Fray et al. (1995); Zhang et al. (2023)
*FcANS, FcUFGT*	Anthocyanin synthesis	Regulate anthocyanin production	Ripe fruits	Figueroa et al. (2021)
*FcPOD27*	Lignin biosynthesis	Promotes lignin production, affecting cell wall rigidity and delaying ripening	Green fruits	Figueroa et al. (2021)

ABA acts as a regulator of the solubilization and depolymerization of polysaccharides. The importance of these polysaccharides, such as pectin, hemicellulose, and cellulose, lies in the fact that they form a supramolecular network that ensures the mechanical support and firmness of the fruit. Castro et al. (2021b) demonstrate that the basis of this molecular regulation lies in the presence of specific cis-regulatory elements, such as ABA-responsive elements (ABRE) and HD-ZIP–type elements, located in the promoter regions of structural cell wall genes, such as polygalacturonase (*FaPG1*), alpha-expansin 2 (*FaEXPA2*), endoglucanase (*FaEG1*), and xyloglucan endotransglycosylase (*FaXTH1*).

The activation of these genes results in a coordinated increase in transcript abundance and in the enzymatic activity of PG, CEL, and XET, which carry out pectin solubilization and depolymerization of the xyloglucan–cellulose network. The biological importance of this process lies in the remodeling of cell wall architecture, in which ABA accelerates the fragmentation of hemicelluloses, transforming highly ordered and compact polymers into shorter chains and low-molecular-weight fragments (Castro et al. 2021b). Notably, although the macroscopic loss of receptacle firmness may not be statistically detectable at very early stages, the internal structural stability is already compromised due to the accelerated degradation of these polysaccharides. Thus, strawberry softening is the final result of a profound physicochemical reorganization induced by ABA, which compromises the mechanical integrity of the fruit in favor of ripening progression.

**Fig. 3:**
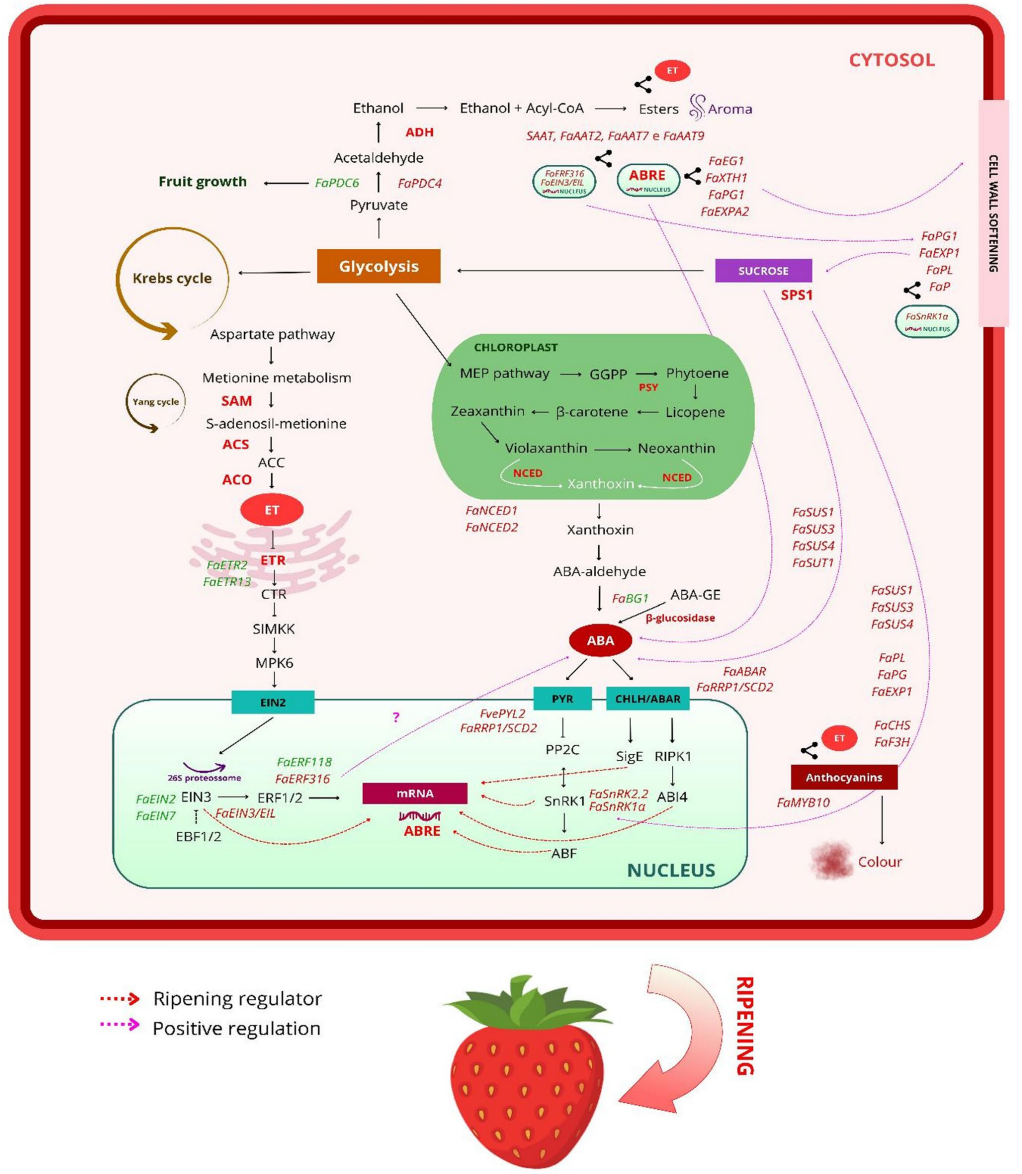
Biosynthesis and signaling pathways associated with strawberry fruit ripening, highlighting the roles of ethylene (ET) and abscisic acid (ABA). ET is synthesized from S-adenosyl-L-methionine (SAM) via the sequential action of ACC synthase (ACS) and ACC oxidase (ACO), and is perceived by ethylene receptors (ETRs; *FaETR2*, *FaETR3*). This perception activates the transcription factors EIN2 and EIN3/EIL, which regulate ripening-related genes such as *FaERF118* and *FaERF316*. ABA is produced through the methylerythritol phosphate (MEP) pathway of carotenoids, involving 9-cis-epoxycarotenoid dioxygenases (*FaNCED1*, *FaNCED2*), and is perceived by PYR/PYL receptors and CHLH. ABA signaling activates SnRK2 kinases (*FaSnRK2.2*, *FaSnRK1a*) and the transcription factors ABF and ABI4. These hormonal pathways converge to positively regulate key genes involved in ripening (*FaEXP1, FaPL, FaPG*), sucrose metabolism (*FaSUS1–4* and *FaSUT1*), and anthocyanin biosynthesis (*FaCHS*, *FaF3H*). Sucrose is associated with ABA signaling through *FaSUS1, FaSUS3, FaSUS4, FaSUT1, FaSnRK2.2*, and *FaSnRK1a* to promote ripening. Genes such as *FaPL, FaPG, FaEXP1, FaCHS*, and *FaF3H* contribute to fruit softening, while *FaPDC4* mediates aroma biosynthesis. Dashed arrows indicate positive regulation (pink), growth regulation (green), or ripening control (red). Red bold text denotes key enzymes in the ripening process, whereas black text indicates substrates

The biosynthesis of ABA in plants begins with the cleavage of β-carotenes, such as 9’-cis-violaxanthin and 9’-cis-neoxanthin, into xanthoxins by the enzyme 9-cis-epoxycarotenoid dioxygenase (*NCED*). This enzyme is crucial for ABA synthesis. During strawberry fruit ripening, *NCED* expression increases along with ABA accumulation. Overexpression of *FaNCED1* (Fig. 3) raises ABA levels and accelerates ripening, while silencing *FaNCED1* lowers ABA levels and delays ripening, affecting sugar accumulation, anthocyanin biosynthesis, scent production, and water conservation (Li et al. 2022). Studies involving the silencing of *FaNCED1* in strawberries indicate a decrease in endogenous ABA levels, which correlates with inhibition of cell wall degradation, demonstrating that repression of this enzyme delays fruit ripening. Additionally, the application of sucrose (50 mM) significantly induced *FaNCED1* expression within 0.5 h, suggesting a rapid response. Conversely, sucrose did not significantly alter the expression of *FaBG1*, which encodes β-glucosidase involved in ABA synthesis by hydrolyzing ABA glucose ester to release active ABA. However, glucose increased the expression of this gene, indicating that sucrose primarily influences the *FaNCED1* pathway (Jia et al. 2013a).

The ABA pathway is initiated when it is perceived by the PYR/PYL/RCARs receptor family, which then interacts with phosphatases (PP2C) that act as negative regulators of ABA. This mechanism, known as the ‘gate-latch-lock mechanism’ involves ABA binding to its receptors (PYR/PYL/RCARs), enabling them to combine with PP2Cs. This interaction results in the removal of PP2C repression on *SnRK2* (Sucrose non-fermenting Related Kinase) activity (Li et al. 2022).

Subsequently, the activated *SnRKs* phosphorylate ABA-responsive transcription factors (ABF) and abscisic acid-insensitive proteins (ABI), activating ABA-responsive genes to trigger physiological responses such as stomatal opening, seed dormancy, and fruit ripening. A second signal transduction pathway reported involves the putative ABA receptor (ABAR) and WRKY transcription factors (WRKY40/ABI5), which acts as a suppressor of ABA signaling (Raghavendra et al. 2010). In strawberries, a clathrin-related protein (*FaRRP1/SCD2*) acts as a key regulator of ripening, linking vesicular transport to ABA signaling. This protein interacts with ABA receptors (*FaPYL2* and *FaABAR*), modulating their affinity for the hormone and activating critical ripening pathways.

The interaction is facilitated by clathrin-mediated endocytosis, aiding ABA absorption and triggering physiological changes in the fruit. Molecular analyses, such as RT-qPCR, showed increased expression of *FaRRP1*, *FaPYL2*, and *FaABAR* in later stages of ripening, and overexpression experiments confirmed their contribution to color and softening. The proposed model suggests that *FaRRP1* integrates the ABA-PYL2-ABI-SnRK2.6 and ABA-ABAR-RIPK1-SnRK2.6 pathways, connecting hormonal transport to ripening control. Although studies on this topic are limited, it may be directly related to sugar metabolism, as clathrin-mediated endocytosis contributes to ABA transport in cells, promoting hormonal responses that include the modulation of carbohydrate metabolism and fruit sensory quality (Li and Shein, 2023).

This connection is further supported by the enzymatic interaction observed in transiently overexpressed *FaSnRK1α* fruits, which leads to the up-regulation of *FaSUS1*, *FaSUS3* and *FaSUS4* (sucrose synthase), *FaPG* (polygalacturonase), *FaPL* (pectate lyase), *FaEXP1* (expansin), *FaCHS* (chalcone synthase), *FaCHI* (chalcone isomerase) and *FaF3H* (flavanone-3-hydroxylase), enzymes linked to pigmentation, cell wall expansion and degradation and sucrose synthesis, thereby enhancing ABA signaling and promoting fruit ripening (Fig. 3) (Luo et al 2020a, b). There is strong evidence for the cascade mechanism of ABA and sucrose acting together on ripening. Additionally, overexpression of the sucrose transporter *FaSUT1* in strawberries can increase the relative expression of key maturation genes such as *BG1, NCED, CHS, PG, PL, SPS* (sucrose-phosphate synthase), and SS, which are crucial for fruit maturation (Jia et al. 2013a). The association of ABA and sucrose with stress ripening can be mediated by *ARS* (ABA-stress ripening). This transcription factor can bind the hexose transporter (HT) promoter, activating sucrose-responsive gene expression, and consequently, inducing maturation of the strawberry receptacle (Jia et al. 2016; Ayub et al. 2016). Siebeneichler et al. (2020), studied the effects of exogenous applications of sucrose and ABA in vivo matured fruits and postharvest matured fruits. Although strawberry is a non-climacteric fruit, there is an induction of red coloration during postharvest maturation due to increased anthocyanins levels and the water deficit caused by the removal of the strawberry fruit from the plant in the white stage. Application of 270- and 500-mM sucrose increases total soluble solids concentration, initiates color change one day after application, and induces postharvest ripening after three days. Treatment with 100 µM of ABA provides higher content of endogenous ABA, ABA-GE (ABA-glycosyl ester—ABA deactivated), proline, and color change after two days.

Proline is an amino acid that responds to stress conditions, which, like sucrose and polyols, contribute to maintaining water balance and the preserving the integrity of proteins, enzymes, and cell membranes (Monteiro et al. 2014; El Moukhtari et al 2020). This is particularly relevant as ABA is an indicator of tolerance to abiotic stresses such as low water availability, regulating stomatal closure to reduce cellular respiration and consequently water loss, thus delaying postharvest ripening longer than sucrose-treated fruits. Evidence supporting these findings includes the application of NDGA (nordihydroguaiaretic acid—NCED inhibitor) in strawberry fruits, which results in reduced total soluble solids, anthocyanins, ABA levels, and a delay in pulp color change (Siebeneichler et al. 2020).

Analysis of brassinosteroid transcription factors revealed that both the negative regulator *FaBIN2* and the transcription factor *FaBZR1* exhibited expression peaks during the pink stage of strawberry development in both field and postharvest assays. Results indicated that exogenous epibrassinolide application influenced the expression of these genes primarily at the pink stage and subsequently, with maximum expression observed 24 h after treatment in the field and 4 h postharvest. This transcription factor expression positively correlated with increased soluble solids and pH, suggesting brassinosteroid involvement in regulating sugar metabolism and acidity during fruit ripening (Ayub et al. 2017).

The biosynthesis of the characteristic aroma of ripe strawberries is a complex metabolic process in which volatile esters are the most important quantitative and qualitative components. The formation of these compounds depends directly on the availability of precursors, primarily alcohols and acyl-CoA molecules. In this context, the pyruvate decarboxylase (PDC) gene family plays a fundamental role by catalyzing the conversion of pyruvate into acetaldehyde and carbon dioxide, the initial step of alcoholic fermentation (Song et al. 2015; Wang et al. 2017). This process leads to the formation of ethanol, an important precursor for the synthesis of the volatile esters responsible for the characteristic aroma and flavor of the fruit.

The availability of pyruvate for this pathway is indirectly related to sugar metabolism, since sucrose is degraded into glucose and fructose, which fuel glycolysis and result in the production of this substrate for PDC activity (Hormazábal-Abarza et al. 2024). During ripening, genes of the *FaPDC* family exhibit specific expression patterns, with some members, such as *FaPDC6*, being more active in the early stages of fruit development, while others, like *FaPDC4*, show higher expression in the late stages of ripening, being associated with the production of aromatic compounds (Hormazábal-Abarza et al. 2024).

The close correlation between the expression profiles of PDC genes and genes of the alcohol acyltransferase (AAT) family suggests that these metabolic pathways are under coordinated genetic regulation during fruit ripening (Aharoni et al. 2000). AATs catalyze the final step of ester biosynthesis by transferring an acyl group from an acyl-CoA donor to an alcohol acceptor, which is frequently derived from PDC activity or other metabolic pathways (Aharoni et al. 2000; Saez et al. 2024). Among the members of this gene family, *SAAT*, *FaAAT2*, *FaAAT7*, and *FaAAT9* stand out, as their transcript levels increase significantly in the late stages of ripening, correlating with total enzymatic activity and the production of aromatic esters (Saez et al. 2024).

This metabolic network is finely modulated by hormonal signals and transcription factors. Strawberry ripening is primarily triggered by an increase in ABA levels and a reduction in auxins within the fruit receptacle (Medina-Puche et al. 2014; Saez et al. 2024). Furthermore, *FaPDC* gene promoters contain ABA-responsive elements (ABREs), indicating that the expression of these genes may be regulated by the natural increase of this hormone during ripening, especially at stages such as the white stage (Medina-Puche et al. 2014; Saez et al. 2024; Hormazábal-Abarza et al. 2024). Concurrently, ABA activates the transcription factor *FaMYB10*, which plays a central regulatory role in secondary metabolism and fruit quality. *FaMYB10* influences volatile composition by regulating genes such as *FaEOBII*, which controls the phenylpropanoid pathway responsible for floral aromas and volatiles like eugenol. Thus, aroma biosynthesis in strawberries integrates primary sugar metabolism, ABA hormonal signaling, and the coordinated gene regulation of PDCs and AATs to ensure that fragrance production occurs synchronously with optimal ripening (Aharoni et al. 2000).

### Revisiting the climacteric and non-climacteric paradigm in strawberry

As a model fruit species, strawberry ripening has been extensively investigated, and its conceptual classification has evolved from being regarded as strictly non-climacteric to a more nuanced view recognizing that ET plays a coordinated and biologically relevant role in its development (Paul et al. 2011). Traditionally, strawberry has been defined as non-climacteric due to its low ethylene production and the apparent inability of exogenous treatments to accelerate ripening in harvested fruit (Perkins-Veazie et al. 1995; Reis et al. 2020). However, in planta monitoring has revealed that at the red-ripe stage the fruit exhibits an autocatalytic increase in ethylene production followed by a respiratory CO₂ climacteric, which under classical physiological criteria could support its classification as climacteric (Iannetta et al. 2006). This shift in interpretation suggests that the categorical designation of strawberry as non-climacteric may be overly simplistic, as it fails to fully capture the fruit’s biological complexity under natural conditions.

Although Iannetta et al. (2006) confirmed this physiological profile, ripening of the strawberry receptacle involves a complex hormonal network predominantly regulated by ABA. As previously discussed, ABA functions as a central coordinator of key physicochemical changes, including tissue softening, pigment accumulation, and modulation of soluble solids and acidity. Moreover, accumulating evidence indicates that ethylene exerts a significant modulatory effect, acting in close interaction with ABA throughout this process (Jia et al. 2011; Elmi et al. 2016; Zhang et al. 2023; Perotti et al. 2023).

Achenes substantially contribute to total ethylene production in ripe fruit and represent the primary site of hormone action in regulating flavonoid biosynthesis, storage compound accumulation, and metabolites associated with final achene coloration (Iannetta et al. 2006; Elmi et al. 2016). In contrast, within the receptacle, ethylene modulates the expression of genes related to cell wall remodeling, such as pectin lyase (*FaPLA*) and polygalacturonase (*FaPG2*), as well as genes encoding intermediates of the tricarboxylic acid cycle, thereby directly influencing respiration, texture, and tissue softening (Merchante et al. 2013). ET biosynthesis begins with methionine, converted to SAM by SAMS, and subsequently to ACC by ACS enzymes, culminating in the production of ethylene via ACO (Pattyn et al. 2021). This signal is perceived in the endoplasmic reticulum by receptors such as *FaETR2* and *FaETR13*, which trigger a cascade involving the disinhibition of EIN2 and the stabilization of EIN3/EILs transcription factors in the nucleus (Figueroa et al. 2021). The functional relevance of this localized ethylene production is supported by molecular evidence. Silencing genes involved in ethylene biosynthesis (*FaSAMS1*) or signaling (*FaCTR1*) results in fruit that fail to develop normal red coloration and exhibit impaired softening, demonstrating that although ethylene does not serve as the primary regulator of ripening rate, it is essential for proper coordination of strawberry maturation (Sun et al. 2013).

At the molecular level, this ethylene dependency reflects the presence of a highly structured signaling network integrated with other hormonal pathways. The identification of an extensive signaling machinery, including 15 *FaETR* receptors and 14 *FaEIN3/EIN* transcription factors, indicates a redundant and sophisticated system integrated with ABA signaling to regulate senescence and sensory quality (Zhang et al. 2022, 2023). Ethylene has been shown to stimulate ABA biosynthesis, as exogenous ET treatments increase ABA levels in strawberry fruit (Elmi et al. 2016). Zhang et al. (2023) further demonstrated that expression of *FaETR2*, *FaETR13*, *FaEIN2*, and *FaEIN7* is induced by ABA and suppressed by NDGA, supporting crosstalk between ethylene and ABA signaling during ripening. Similarly, Chen et al. (2023) reported that ethephon treatment enhances ABA biosynthesis by activating *FaNCED1* in postharvest strawberries.

Ethylene effects in strawberry are not uniform but depend strictly on developmental stage and physiological context, namely whether fruit remain attached to the plant or are harvested. At early developmental stages, such as large-green fruit, ethylene exerts an unexpected regulatory function. In *Fragaria chiloensis*, ethephon application at this stage represses anthocyanin biosynthesis by redirecting carbon flux within the phenylpropanoid pathway. This response involves downregulation of key pigmentation genes (*FcANS* and *FcUFGT*) and upregulation of lignification-related genes such as *FcPOD27*, leading to increased lignin accumulation at the expense of red coloration (Figueroa et al. 2021).

In contrast, fruit responses to ethylene during ripening differ markedly depending on experimental conditions. In postharvest fruit, ethylene treatment accelerates senescence-related processes, enhancing sucrose hydrolysis, increasing respiration, and reducing acidity and firmness (Figueroa et al. 2021). However, when fruit are treated while still attached to the plant, ethylene does not function as a ripening trigger. Under these conditions, although ethephon may elevate anthocyanin content particularly when applied at the white stage and alter volatile (ester) profiles, it does not accelerate visible ripening or significantly modify sugar and soluble solids content (Reis et al. 2020). This discrepancy between attached and detached fruit may be explained by the integrity of the source–sink relationship. In attached fruit, sugar accumulation depends on continuous photoassimilate transport from leaves, whereas in detached fruit this supply is interrupted. Consequently, postharvest increases in sugars may reflect stress responses or cell wall degradation rather than standard physiological ripening (Iannetta et al. 2006; Reis et al. 2020). Thus, in strawberry, ethylene operates primarily as a stage-specific metabolic modulator rather than as the primary ripening signal (Reis et al. 2020).

Despite its multiple roles, ethylene action in strawberry differs substantially from that observed in classical climacteric fruit. Nevertheless, its physiological behavior shares similarities with fruit classified as suppressed climacteric, in which the autocatalytic ethylene pathway is attenuated but not entirely absent.

In suppressed-climacteric Japanese plums such as ‘Shiro’ and ‘Rubyred’, ethylene production is 15- to 500-fold lower than in fully climacteric cultivars. This phenotype arises from impaired conversion of ACC to ethylene or from limited hormone perception and receptor regeneration in the absence of external stimulation (Abdi et al. 1998). Analogously, strawberry, although historically labeled non-climacteric, displays in planta a linear and continuous rise in ethylene at the red-ripe stage, followed by a CO₂ respiratory peak. However, the absolute amount of ethylene released by strawberry (approximately 1 nL fruit⁻^1^ h⁻^1^) is 20- to 2000-fold lower than that of canonical climacteric fruit such as tomato or apple (Iannetta et al. 2006). At the molecular level, both systems possess extensive ethylene signaling machinery, including ACS and ACO biosynthetic genes and multiple ethylene receptors. In strawberry, the low magnitude of ethylene production is offset by exceptionally high biological sensitivity, whereby minute hormone concentrations suffice to coordinate senescence and aroma development. In both models, transition to the final ripening phase is associated with declining auxin levels, which initially act as repressors of ethylene and ABA pathways (Iannetta et al. 2006; Fuentes et al. 2019; Perotti et al. 2023).

Therefore, the concept of suppressed climacteric may provide a biological framework explaining why exogenous application of ethylene or propylene can accelerate ripening in both suppressed plums and strawberry. This convergence supports the view that traditional distinctions between ripening categories are giving way to a broader understanding of ethylene ubiquity among fleshy fruits (Paul et al. 2011).

Rather than governing ripening velocity per se, ethylene in strawberry primarily modulates secondary metabolism and sensory quality, stimulating the accumulation of free amino acids and essential aroma volatiles. Current evidence thus indicates that strawberry ripening is a finely coordinated process in which ethylene operates within a positive feedback system at late stages, signaling senescence progression, aroma development, and fruit integrity.

## Conclusion and future perspectives

Understanding the hormonal metabolism of fruits paves the way for emerging directions in scientific research and technological advancements aimed at enhancing strawberry quality. Strawberry development and ripening are governed by a highly coordinated hormonal transition that challenges the traditional binary classification between climacteric and non-climacteric fruits. The process is initiated by functional communication between the achenes and the receptacle, where high IAA synthesis in the early stages promotes cell expansion and acts as an active repressor of ripening. The drop in IAA and GA levels acts as the biological trigger needed to deactivate growth genes and allow the activation of ABA and ET pathways, which take over the regulation of late ripening.

Strawberries exhibit behavior that may resemble “suppressed climacteric,” presenting a respiratory peak and an autocatalytic increase in ethylene in the red fruit stage. Although produced in low concentrations, ethylene has exceptional biological sensitivity, acting in synergy with ABA to modulate the synthesis of anthocyanins, volatile aromas, and cell wall degradation. This interaction is reinforced by sucrose, which acts not only as a carbon reserve but also as a signaling molecule capable of rapidly inducing the expression of genes such as *FaNCED1*, accelerating ABA biosynthesis and the ripening cascade.

Given this complexity, it is proposed that future research focus on biochemical crosstalk, such as competition for the GGPP precursor between the gibberellin and ABA pathways, and the relationship between ABA and sugars and ethylene through transcription factors. In addition, further studies on ethylene *in planta* would be interesting to better understand the relationship of this hormone with strawberry ripening, since its fruits are responsive to ethylene and this can redirect important gaps for the development of genotypes with greater pulp resistance and extended post-harvest shelf life.

## Data Availability

No datasets were generated or analysed during the current study.
